# Targeting Aging and Diseases Associated with Ferroptosis and Senescence Through Modulation of Iron, Oxidative Stress and Lipid Peroxidation

**DOI:** 10.3390/antiox15010015

**Published:** 2025-12-22

**Authors:** Malamati Kourti, George J. Kontoghiorghes

**Affiliations:** 1 Postgraduate Research Institute of Science, Technology, Environment and Medicine, 3021 Limassol, Cyprus; m.kourti@euc.ac.cy; 2 Department of Life Sciences, European University Cyprus, 2404 Nicosia, Cyprus

**Keywords:** ferroptosis, senescence, iron, chelators, oxidative stress, aging, senolytics, senomorphics

## Abstract

Ferroptosis and senescence are unique cellular processes that lead to irreversible cell abnormalities and tissue damage in many diseases, such as cancer, neurodegeneration, cardiac, liver, and kidney damage. Despite distinct differences between the two processes, essential shared features in their causes and development include increased redox iron toxicity and oxidative stress, together with reduced antioxidant capacity, such as decreased glutathione levels and downregulation of glutathione peroxidase. The consequences of these toxicities include increased lipid peroxidation and aggregation, causing cell damage and death in ferroptosis, whereas in senescence, they lead to DNA and other biomolecular damage, resulting in a form of cell growth arrest with specific characteristics, such as the progressive accumulation of senescent cells across tissues in aging. Many potential therapeutic strategies have emerged to regulate ferroptosis and senescence pathways, including targeting and modulating iron toxicity and redox imbalance, and metabolic, transcriptional, genomic, and other associated pathways and factors. Experimental evidence suggests that iron chelating drugs such as deferiprone, deferoxamine, and deferasirox, and other drugs such as sorafenib, may be potential therapeutics for ferroptosis. Similarly, in senescence, in addition to iron chelating drugs that can act as senomorphic and senolytic agents, several other drugs, such as navitoclax and the combination of dasatinib and quercetin, have shown promising results in preliminary clinical trials as senolytic agents, while rapalogs and several nutraceuticals, such as quercetin, have been studied as senomorphic agents. Despite the absence of antioxidant drugs in clinical practice, the development of therapeutic strategies, including the repurposing of iron chelating drugs and the use of natural antioxidants, may be crucial for therapeutic advances in diseases associated with ferroptosis and senescence. The design of new therapeutic strategies based on the modulation of multiple targets, particularly the control of redox iron and oxidative stress toxicity using combinations of iron chelators with other drugs or nutraceuticals, may improve therapeutic outcomes in many diseases associated with ferroptosis, senescence, and aging. In each case, target selection and specific considerations may apply within the context of personalized medicine.

## 1. Introduction

Ferroptosis and senescence are unique processes of cellular damage, which are found to be associated with many different diseases, including cancers, organ damage, neurodegeneration, and aging. In both ferroptosis and senescence, some of the critical or important toxicity pathways leading to cellular damage appear to involve iron metabolism deregulation and redox toxicity, oxidative stress, and biomolecular damage, such as lipid peroxidation. In this context, a better understanding of the toxicity pathways involved and their modulation or control could eventually lead to the treatment of many diseases associated with ferroptosis and senescence, which affect millions of patients worldwide.

Oxidative stress, which is the imbalance between cellular pro-oxidant agent production, such as reactive oxygen/nitrogen species (ROS/RNS), and antioxidant capacity, is a common upstream driver that can lead to the pathogenesis and damaging effects of many diseases, including both organismal aging and numerous age-associated pathologies [1,2,3]. The production of ROS is a normal physiological process, which is controlled by an antioxidant system involving enzymes, endogenous antioxidants, and nutrients. At low levels, ROS can function as signaling molecules, but chronic and excessive ROS production can cause cumulative biomolecular damage, including damage to DNA, lipids, proteins, and sugars, damage to mitochondrial function, and activation of stress signaling that can impair tissue biomolecular homeostasis and structural integrity [1,2,3,4,5]. Importantly, oxidative stress is not just a direct cause of damage, but it can also trigger and reinforce cellular changes, such as mitochondrial dysfunction and inflammation, that can lead to a self-perpetuating state, which is harmful to tissue function and repair [1].

A major component required for the maintenance of redox homeostasis in cells is the presence and control of iron, which is required for many other physiological functions [6]. In particular, iron interconnects redox chemistry and cellular fate mainly because its redox-active ferrous form (Fe^2+^) catalyzes Fenton reactions to generate highly reactive hydroxyl radicals (·OH) that particularly promote, among others, lipid peroxidation and oxidative DNA damage [1,2,3]. Thus, deregulated iron metabolism, including increased iron uptake, malfunction of related proteins, impaired storage/export, aberrant ferritin turnover, and other abnormalities, could increase oxidative burden and convert otherwise controlled redox signaling into pathological oxidative stress [2,3,6,7,8]. This iron-driven amplification of ROS explains why iron homeostasis is central to both ferroptosis and senescence and is a primary therapeutic target for diseases where redox balance is disrupted.

From a translational point of view, the link between iron, oxidative stress, senescence, and ferroptosis can be best understood in terms of how long the stress lasts and how the cell responds. For example, persistent, sublethal oxidative stress promotes permanent cell-cycle arrest and a pro-inflammatory, tissue-remodeling secretome (the senescence-associated secretory phenotype or SASP) that spreads dysfunction across cells and organs, a process central to senescence, aging, and many chronic diseases [1,9]. In contrast, in almost all diseases where iron-catalyzed lipid peroxidation overwhelms local antioxidant defenses, the same redox chemistry can initiate in specific cell types an acute, lytic form of programmed cell death called ferroptosis [10,11,12]. Both ferroptosis and senescence are linked to oxidative chemistry but differ in kinetics, downstream tissue effects, and therefore therapeutic opportunities.

Because iron is a direct initiator and amplifier of harmful oxidative chemistry in biological systems, the control and modulation of redox activity by iron is a rational and clinically valid target for therapeutic strategies involving pathologies associated with free radicals [13,14,15]. In particular, the inhibition of iron toxicity, including damage related to Fenton oxidative reactions, can be achieved using naturally occurring and synthetic iron chelators [13,14,15]. Most importantly, the widely used iron chelating drugs deferoxamine (DF), deferiprone (L1), and deferasirox (DFRA) are known to inhibit Fenton reactions, lower labile iron pools, and have favorable effects on tissue oxidative markers [13,14,15,16,17,18,19]. Furthermore, experimental and early clinical data suggest benefits of iron chelation therapy, including targeted modulation of ferritinophagy and ferroportin pathways in the context of diseases associated with free radical pathologies, such as neurodegeneration, cancer, and organ injuries [13,14]. At the same time, iron-targeting strategies must be precise, effective, and non-toxic. In this context, reducing labile redox-active iron levels by chelation can protect cells from ferroptotic death and mitigate senescence-driving oxidative stress, while not interfering with essential iron-dependent physiology or causing serious chelator-related toxic side effects [13,14,19]. These considerations support the design of specific strategies aimed at exploiting iron and oxidative stress modulation to both prevent maladaptive senescence and selectively manipulate ferroptotic susceptibility in disease.

In summary, oxidative stress and its relationship with iron provide the link between the chronic, survival-oriented program of senescence and the acute, death-oriented cascade of ferroptosis. At the same time therapeutic strategies such as iron chelation may offer opportunities for the treatment of many diseases of free radical pathologies associated with ferroptosis and senescence. The main purpose of this review is to identify the mechanisms and implications of iron deregulation and oxidative stress in ferroptosis and senescence in relation to major diseases affecting millions of people worldwide. Furthermore, it aims to identify targets and suggest new approaches and strategies with therapeutic applications, including the use of iron chelating drugs and other drugs with antioxidant properties, which could help in the treatment of different diseases associated with ferroptosis and senescence.

## 2. General Aspects of Iron Metabolism

Iron is one of the essential metal ions required for the growth and proliferation of all types of cells, including normal, microbial, and cancer cells. There are many variations among the different types of cells regarding the requirements for iron, iron storage capacity, and different susceptibility to iron toxicity levels. Under normal physiological conditions, iron metabolism in humans is strictly controlled and regulated by specific proteins involved at all the stages of iron absorption, transport, distribution, storage, and utilization [20,21,22,23,24]. Similarly, iron metabolic balance in humans is achieved and maintained due to the presence of specific metabolic pathways, involving proteins and transcription factors, which have evolved for the uptake, distribution, utilization, recycling, and excretion of iron [20,21,22,23,24]. There are many diseases associated with iron metabolic imbalance, including iron deficiency, which affects one in four individuals in the world, and iron overload, which affects millions of people due mainly to genetic abnormalities, such as primary hemochromatosis and secondary hemochromatosis, which is caused by regular transfusions, such as thalassemia major [6,23,24,25,26]. In general, iron overload is a negative prognostic factor for all diseases [8,26].

### 2.1. Iron Absorption and Other Iron-Related Metabolic Pathways

Iron balance in humans is maintained when body iron intake is equivalent to iron excretion, plus other losses of iron from the body [27]. The absorption of iron from different dietary sources takes place in the gastrointestinal tract, with the enterocyte playing a major role in its regulatory control and balance. The general mechanism of iron absorption involves several steps, including initially the conversion of ferric iron (Fe^3+^) to ferrous iron (Fe^2+^) through the activity of a ferroreductase protein present at the cell surface of the enterocyte and the intracellular transport of iron into the enterocyte via the apical divalent metal transporter protein 1 (DMT1) [6,22,28,29]. The absorbed iron is then transferred into the low-molecular-weight transit iron pool and into ferritin of the enterocyte. The rate of transfer of the absorbed iron from the enterocyte into plasma is primarily regulated by the transmembrane protein ferroportin in conjunction with the protein hormone hepcidin. In this context, hepcidin can bind ferroportin, causing its internalization and degradation within the enterocyte, thus inhibiting the release of iron into plasma [29,30,31,32]. Iron trapped in the enterocytes and not released into plasma returns in the gut lumen following shedding of the enterocytes, which occurs every few days. In contrast, the export of iron from enterocytes by ferroportin in plasma and its uptake by transferrin results in the transfer of iron to all the cells of the body [21,22,24,29]. Similar control of iron release into plasma by hepcidin is also observed in other cell types such as macrophages [21,29,30,31,32].

Hepcidin appears in general to affect the overall rate of iron distribution in the body, including the transfer of iron to the hematopoietic tissues. However, hepcidin function abnormalities could also be observed, which could lead to iron metabolic imbalance and abnormal body distribution, including iron overload and the anemia of chronic disease [33]. In addition to the effects of hepcidin, iron absorption and distribution could also be affected by changes in transcription and other factors related to the expression of ferroportin and DMT1, which are expressed differently in various organs such as the liver, duodenum, hematopoietic tissues, and kidneys [34,35,36]. Several other factors, including chelating dietary molecules and drugs, could also affect the absorption and distribution of iron in the body and in different cell types [13,14,15,24,29]. Overall, it appears that different regulatory molecules, transcription factors, dietary molecules, and drugs influence the uptake of iron from the gastrointestinal tract and its distribution to different organs and cells of the body [13,14,15,21,22,23,24,29,30,31,32,33,37].

There are many other abnormalities related to iron metabolism resulting in iron metabolic imbalance, which can be caused by different genetic, dietary, pharmacological, environmental, and other factors, and different diseases, aging, abnormal organ function, sports activities, excessive bleeding, and other changes [27,29,38,39,40,41,42,43,44,45]. In most of these changes, pharmacological intervention, such as iron supplements, is required to restore iron balance. In other cases, for example, body iron reductions due to blood loss in blood donors or small traumas, or long-distance runners, iron balance is gradually restored from increased gastrointestinal intake of dietary iron [39,46].

### 2.2. Proteins of Cellular Iron Transport, Storage, and Utilization

Molecular iron in biological systems is mainly found in the positively charged ferric (Fe^3+^ or Fe (III)) or ferrous (Fe^2+^ or Fe (II)) forms [24]. Ferric iron is sparingly soluble in aqueous solutions at physiological pH. Under these conditions, ferric iron progressively precipitates, forming insoluble polymeric complexes, and only trace amounts of soluble iron are detectable in solution [24]. Ferrous iron is more soluble than ferric iron but is rapidly oxidized to the latter at physiological pH. In contrast, at acidic pH, such as the acidic environment of the stomach or in lysosomes, the solubility of iron increases. Similarly, the solubility of iron increases in the presence of iron-binding ligands and especially specific chelators, which can form soluble iron complexes [24]. In particular, iron in biological systems is always found bound to different ligands present in different biomolecules, ranging from low-molecular-weight ones, such as citrate, to high-molecular-weight proteins, such as transferrin [24,47,48].

At the cellular level, the requirements and utilization of iron differ for each cell type and mostly depend on the fulfillment of specific biological functions and on the ability to store and maintain different quantities of iron [20,21,22,23,24]. The basic metabolic pathway of cellular iron uptake and storage involves a series of steps. In particular, the transfer of iron in blood and its donation to all the cells of the body is carried out by the iron transport protein transferrin, which has two iron chelating sites and can bind a maximum of two molecules of ferric iron (Fe^3+^), one in each site. The intracellular transfer of iron primarily involves diferric transferrin uptake through transferrin receptors, which are present on the cell membrane [24,49,50,51,52,53]. Following endocytosis of the diferric transferrin/transferrin receptor complex, iron is released due to the acidic pH of the endosome into the intracellular low-molecular-weight transit iron pool composed of low-molecular-weight natural chelators and iron [48,53]. This transit iron pool is utilized in the cell for the turnover of iron-containing proteins or for transfer of iron into ferritin for iron storage [24,48].

Ferritin is a hollow protein sphere found in all cells. One molecule of ferritin can store up to a maximum of 4500 molecules of ferric iron in the form of ferric oxyhydroxide phosphate complexes [54,55,56,57]. Intracellular iron storage can also be found in the form of hemosiderin, which is a cluster of ferritin molecules with a broken protein shell and exposed iron deposits [24,58]. Hemosiderin is at a low concentration in normal individuals but predominates over ferritin in patients with heavy iron-loaded clinical conditions [24,59,60,61]. Variation in the sites of iron deposition and iron storage capacity is observed in each organ of different clinical conditions [62,63]. For example, in most cases of chronically red blood cell transfused patients with thalassaemia, excess iron is predominantly stored in the liver and to a lesser extent in the spleen, heart, and other organs [62,63].

There are many other iron-binding or -containing proteins playing an important role in iron metabolism. For example, lactoferrin, the sister protein of transferrin found in bodily secretions and neutrophils, is also a powerful natural iron chelator and antioxidant with antimicrobial and many other physiological activities, similar to transferrin [64,65,66,67]. Similarly, there are many other iron-containing proteins such as oxygenases, reductases, hydrolases, and hydrogenases playing important roles in cellular functions, including energy transduction, oxygen storage and transport, DNA synthesis, and lipid metabolism [24].

### 2.3. The Role of Iron in Redox Cycling and the Effects of Chelators

One of the major roles of iron in biological systems is redox cycling, which under certain conditions may also cause redox iron toxicity, including oxidative damage in all biomolecules [2,3,8,16,17,18,68,69,70]. In particular, iron plays an important catalytic role in the production of free radicals, including its participation in metalloenzymes, in metalloproteins of mitochondria, and as low-molecular-weight iron complexes, some of which are involved in the Fenton reactions [2,16,17,18,24,68,69,70]. Overall, iron appears to play a crucial role in the maintenance of redox homeostasis.

Iron modulation and inhibition of iron catalytic activity in the Fenton reactions constitute major targets in many diseases associated with free radical pathologies. In this context, natural and synthetic iron chelators and especially iron chelating drugs could be used in many conditions for targeting increasing oxidative damage arising from Fenton reactions related toxicity in many diseases, including those associated with ferroptosis and senescence [13,14,15,16,17,18,19]. Furthermore, other related targets against oxidative damage may include antioxidants and other inhibitors of oxidative stress, inhibition of key proteins and their membrane receptors, intracellular iron transport and extracellular metabolic pathways, different organelles and other cellular compartments, genomic, transcriptional, and other factors [68,69,70].

In general, natural and synthetic chelators, which could be used to mobilize low-molecular-weight labile iron, may potentially inhibit iron-catalyzed free radicals and ROS and associated processes of oxidative stress toxicity and damage, including pathways such as lipid peroxidation in ferroptosis. Other antioxidants, such as vitamin E, could also minimize oxidative stress toxicity and inhibit pathways related to ferroptosis and senescence, but cannot inhibit the initiation of free radicals, ROS, and related cascades catalyzed by iron.

Many different pharmacological approaches would be needed in each disease associated with either senescence or ferroptosis. For example, pharmacological aspects such as drug administration period and selected posology are likely to be relatively of short-term duration in ferroptosis associated with cancer and long-term duration in senescence associated with aging [71]. Targeting aspects and the selection of appropriate drugs are also important pharmacological parameters associated with either senescence or ferroptosis. For example, all three iron chelating drugs (DF, L1, and DFRA) are effective in mobilizing labile iron and inhibiting Fenton reactions at variable levels, but their pharmacological, toxicological, and organ targeting properties are different, and subject to risk/benefit assessment in each case [13,14,15,16,17,18,19].

## 3. Ferroptosis

### 3.1. Main Mechanisms of Ferroptosis

Ferroptosis is a regulated, programmed form of cell death, first defined in 2012, which is characterized by the iron-dependent accumulation of lipid peroxides, and it is distinct from other forms of cell death, such as apoptosis, necroptosis, and autophagy [10]. Unlike apoptosis, necroptosis, or autophagy, ferroptosis is executed through metabolic failure rather than caspase activation or classical membrane rupture [10]. Its initiation and progression depend on the interplay of three molecular hallmarks: (i) the intracellular presence of excess labile iron, (ii) failure of antioxidant defenses, and (iii) uncontrolled lipid peroxidation [11].

**Figure 1:**
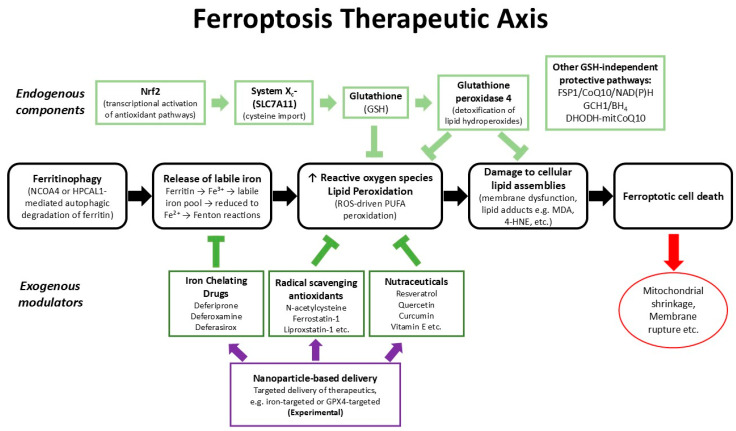
Schematic representation of key pathways regulating ferroptosis and their therapeutic modulation. Ferroptosis is an iron-dependent cell death driven by lipid peroxidation and oxidative stress. It is initiated by inhibition of the System X_c_− antiporter, GSH depletion, and GPX4 inactivation, while ferritinophagy increases the labile iron pool, driving Fenton reactions and lipid damage. Protective mechanisms involve nuclear factor erythroid 2-related factor 2 (Nrf2), which induces antioxidant and iron-sequestering genes, and FSP1/DHODH, which regenerate CoQ_10_ to limit lipid radicals. Therapeutically, ferroptosis inhibitors and iron chelators such as deferoxamine, deferiprone, and deferasirox prevent oxidative damage in different diseases. Complementary strategies include radical-scavenging antioxidants such as ferrostatin-1 and curcumin, nutraceuticals such as resveratrol and quercetin, and nanoparticle delivery systems to enhance precision and safety of these agents. (Color coding and arrows: Black boxes and arrows indicate core molecular events driving ferroptotic signaling. Green boxes and arrows represent endogenous and exogenous antioxidant and iron-regulatory pathways or therapeutic interventions that protect against ferroptosis. Purple boxes and arrows denote advanced therapeutic interventions through different delivery methods. Red arrows/outlines highlight terminal cellular damage and ferroptotic cell death. Abbreviations: BH_4_: tetrahydrobiopterin. CoQ_10_: co-enzyme Q_10_. DHODH: dihydroorotate dehydrogenase. FSP1: ferroptosis suppressor protein 1. GCH1: GTP cyclohydrolase 1. GPX4: glutathione peroxidase 4. GSH: glutathione. 4-HNE: 4-hydroxynonenal. HPCAL1: Hippocalcin-like 1. MDA: malondialdehyde. NCOA4: nuclear receptor coactivator 4. Nrf2: nuclear factor erythroid 2-related factor 2. PUFA: polyunsaturated fatty acid. ROS: reactive oxygen species. SLC7A11: Subunit of the System X_c_− cystine/glutamate antiporter. System X_c_−: Cystine/glutamate antiporter system.).

Iron plays a central role in ferroptosis by catalyzing Fenton reactions and generating ROS, such as hydroxyl radicals, that accelerate peroxidation of polyunsaturated fatty-acid (PUFA)-containing phospholipids on membranes [12]. As discussed in the previous section, intracellular iron availability is controlled mainly by transferrin-receptor-mediated uptake, ferritin storage, and ferroportin export [72]. Deregulation of these pathways can shift the balance towards ferroptotic sensitivity [11]. On the pro-death side, a critical point in the progression of ferroptosis is the increased activity of ferritinophagy, which appears to be mediated through the nuclear receptor coactivator 4 (NCOA4). Recent studies have also identified Hippocalcin-like 1 (HPCAL1) as an additional receptor involved in regulating ferroptotic susceptibility. Similarly to NCOA4, HPCAL1 binds ferritin and promotes its lysosomal degradation, thereby increasing the labile iron pool and enhancing iron-dependent lipid peroxidation. Notably, loss of HPCAL1 reduces ferritin turnover and protects cells from ferroptosis, whereas its overexpression enhances ferroptotic sensitivity through iron mobilization and downstream ROS accumulation [73,74]. These findings suggest that HPCAL1 functions in parallel with NCOA4 as a ferritinophagy receptor and contributes to the initiation phase of ferroptosis through control of intracellular iron availability. Ferritinophagy takes place in lysosomes, where stored polynuclear iron in ferritin is solubilized in the acidic environment of lysosomes and increasingly converted into labile iron. The reduction of iron Fe^3+^ into Fe^2+^ from the increasing labile iron pool appears to fuel excess ROS production via Fenton reactions [75,76]. This excess redox iron amplifies oxidative stress and directly promotes excess lipid peroxidation in membranes and other parts of the cells, and the formation of lipid peroxide conjugates, which develop into large lipid peroxide intracellular assemblies, eventually leading to cellular damage and death (Figure 1) [12,77].

Under normal physiological conditions the antioxidant system involves different enzymes, endogenous antioxidants, and nutrients such as vitamins C and E, all of which can act effectively against oxidative processes and restore redox balance [78]. However, enhancement of the oxidative activity induced by iron in cells during ferroptosis could not be easily reversed. In particular, the selenoenzyme glutathione peroxidase 4 (GPX4), which plays a major role in the intracellular antioxidant activity and is also the principal suppressor of ferroptosis, appears to be insufficiently effective in detoxifying lipid hydroperoxides and reducing them to nontoxic alcohols, usually using glutathione (GSH) as cofactor [79,80]. Similarly, ferroptosis can be triggered by limiting cysteine import via the cystine/glutamate antiporter system X_c_− (SLC7A11), by depleting GSH, or by directly inhibiting GPX4, which disorganizes this protective axis [81]. Beyond GPX4, a supplementary protective pathway, the ferroptosis suppressor protein 1 (FSP1)-coenzyme Q_10_ (CoQ_10_)-NAD(P)H pathway, suppresses ferroptosis independently of GSH by reducing lipid radicals at membranes [82,83]. Additional factors, including the GTP Cyclohydrolase 1 (GCH1)-tetrahydrobiopterin (BH_4_) axis [84] and dihydroorotate dehydrogenase (DHODH) [85], protect specific subcellular compartments by acting as GPX4-independent ferroptosis suppressors, preventing lipid peroxidation.

Another major component affecting ferroptosis is lipid metabolism. In this context, the lipid substrate pool is formed by acyl-CoA synthetase long-chain family member 4 (ACSL4), which enriches cellular membranes with PUFA-derived acyl-CoAs, such as arachidonic acid, that are then esterified into phospholipids [86]. These PUFA-phosphatidylethanolamines are preferentially oxidized during ferroptosis [87]. The iron-containing enzymes lipoxygenases (LOXs) and related oxygenases catalyze the enzymatic peroxidation of PUFA-containing phospholipids, contributing to lethal lipid peroxide accumulation [87,88]. When the burden of lipid peroxides exceeds the detoxifying capacity of antioxidant systems, ferroptotic cell death follows, representing a metabolic “point of no return” where iron handling, redox homeostasis, and lipid metabolism convergence are out of control [11]. Several factors are known to affect the rate of ferroptosis. For example, tumor-suppressive and stress-responsive pathways modulate this threshold, while broader stress signals and metabolic checkpoints also tune susceptibility [89,90].

### 3.2. Diseases Associated with Ferroptosis

Ferroptosis is increasingly recognized as a central mechanism of the cellular death process across various diseases, including cancer, neurodegeneration, organ injury, and cardiovascular diseases, among others, with shared hallmarks of labile/redox active iron toxicity, antioxidant system collapse, and PUFA-driven lipid peroxidation. Some examples of diseases where ferroptosis has been implicated are described below.

#### 3.2.1. Ferroptosis and Cancer

It appears that in each disease, different types of cells are involved, and conditions apply affecting not only the process of ferroptosis but also possible therapeutic interventions. In particular, there is an increasing interest in the role of ferroptosis in different types of cancer, cancer metastasis, and drug resistance [91]. Furthermore, ferroptosis is increasingly recognized as a double-edged process that shapes both tumor development and therapeutic responses [92]. In this context, many cancers display metabolic adaptations that reduce ferroptotic susceptibility, such as upregulation of cystine importers, including SLC7A11 [93,94,95] or altered lipid composition of membranes [96], thereby ensuring survival under oxidative stress. Conversely, certain oncogenic or tumor suppressor pathways remodel ferroptosis sensitivity, influencing tumor progression and heterogeneity. For example, p53 can exert context-dependent effects [97], promoting ferroptosis in some contexts, such as under oncogenic or DNA damage stress, via repression of SLC7A11 [98], but can also suppress ferroptosis through induction of p21 and modulation of lipid metabolism in metabolically stressed conditions [90]. Meanwhile, BRCA1-associated protein 1 (BAP1) links chromatin regulation to ferroptosis by repressing SLC7A11 via histone H2A deubiquitination, thus lowering cysteine uptake [99].

In contrast, exploiting ferroptosis has emerged as a target strategy to destroy cancer cells or overcome cancer therapy resistance [91]. For instance, treatment-refractory tumors often rely on GPX4 activity [100] or alternative antioxidant pathways such as FSP1-CoQ_10_ [81], and pharmacological or genetic induction of ferroptosis may resensitize them to chemotherapy, radiotherapy, or targeted inhibitors [92]. Preclinical studies suggest that ferroptosis induction synergizes with immune checkpoint blockade, as dying ferroptotic cells can release danger signals that enhance antitumor immunity [101]. However, ferroptosis may also promote tumorigenesis under certain conditions by fostering chronic inflammation through the release of lipid peroxidation products, such as 4-hydroxynonenal (4-HNE) or malondialdehyde (MDA) [102]. This dual role underscores the need for precision in harnessing ferroptosis for cancer therapy, targeting it as a vulnerability in established tumors while mitigating its potential pro-tumorigenic consequences in the tumor microenvironment [103].

#### 3.2.2. Ferroptosis and Neurodegenerative Diseases

Neurodegenerative diseases appear to show a concurrent ferroptotic signature in vulnerable brain regions, including iron accumulation, impaired glutathione/GPX4 defenses, PUFA-rich membrane remodeling (often via ACSL4), and excessive lipid peroxidation with release of related toxins, such as 4-HNE and MDA. In Alzheimer’s disease, hippocampal and cortical iron loading accelerates ROS-driven lipid damage [104]. Concomitant GPX4 downregulation and GSH depletion weaken detoxification capacity in the brain [105], while ACSL4-dependent enrichment of oxidizable phospholipids amplifies susceptibility to ferroptosis, consistent with elevated lipid peroxidation adducts in patient tissue and models [106]. Similarly, in Parkinson’s disease, substantia nigra excess iron deposition promotes Fenton reactions. Iron also promotes α-synuclein aggregation [107], which feeds back on redox imbalance and lipid ROS, while reduced levels of GPX4/GSH and increased levels of ACSL4 sensitize dopaminergic neurons [108,109].

The above ferroptotic mechanisms are generally supported across many reviews and experimental systems of neurological diseases. For example, in amyotrophic lateral sclerosis, motor neurons and spinal cord tissue exhibit iron accumulation, GPX4/GSH insufficiency, and heightened lipid ROS [110,111]. Mutant superoxide dismutase 1 (SOD1) further elevates oxidative stress [112], and augmenting GPX4 activity can mitigate motor neuron degeneration in models [113,114], aligning amyotrophic lateral sclerosis’s pathology with ferroptosis biology [115]. Huntington’s disease similarly shows iron deregulation in basal ganglia/cortex, GPX4 downregulation, and lipid peroxidation-linked mitochondrial dysfunction [116,117], with emerging evidence that ferroptosis contributes to neuronal toxicity and disease progression [118,119]. Finally, multiple sclerosis features iron deposition within chronic active lesions and microglia, increased lipid ROS and oxidative stress that damage oligodendrocytes, and decreased GPX4 in gray matter and experimental autoimmune encephalomyelitis models [120,121,122]. The introduction of pharmacologic ferroptosis inhibition, for example, with the iron chelating drug L1 [123], attenuates experimental disease, implicating ferroptosis in neuroinflammation and demyelination.

#### 3.2.3. Ferroptosis and Kidney Diseases

Ferroptosis is also increasingly recognized as a decisive driver of epithelial damage in kidney diseases, such as Acute Kidney Injury (AKI), whether induced by ischemia–reperfusion, nephrotoxins, or rhabdomyolysis [124]. In these models, well-documented ferroptotic mechanisms participate in renal damage. For example, excess iron liberated via NCOA4-mediated ferritinophagy and other routes, catalyzes Fenton reactions leading to lipid peroxidation and tubular cell death [125,126]. Concurrently, depletion of GSH and inactivation of GPX4 remove critical antioxidant defenses, making renal cortex membrane lipids highly vulnerable [126]. Notably, p53 is activated under tubular stress conditions, such as folic-acid-induced AKI, sensitizing renal epithelial cells to ferroptosis, a process that can be attenuated by antioxidants like α-lipoic acid [14,127]. Moreover, as described above, the GPX4-independent FSP1-CoQ_10_-NAD(P)H axis serves as a backup defense, which, when impaired, intensifies ferroptotic injury also in kidney diseases.

Recent interventions have been shown to suppress aberrant ferritinophagy and relieve ferroptosis via modulation of lysosomal function in ischemia–reperfusion AKI models [128]. Together, these findings highlight ferroptosis as a central, targetable node in AKI pathophysiology.

#### 3.2.4. Ferroptosis and Cardiovascular Pathologies

Ferroptosis contributes significantly to various cardiovascular pathologies, especially myocardial ischemia–reperfusion injury, myocardial infarction, and cardiomyopathy. In these myocardial pathologies, myocardial tissue demonstrates sustained ferritinophagy and iron release via NCOA4, overwhelming antioxidant systems. Reduced GPX4 and GSH levels are common, while elevated ACSL4 and LOX, such as ALOX15 activity, accelerate PUFA-phospholipid peroxidation [129]. In atherosclerosis and vascular injury, endothelial ferritinophagy promotes labile iron accumulation, and LOX-driven lipid oxidation destabilizes plaque and impairs endothelial function [130]. Cardiac hypertrophy and heart failure feature iron-driven lipid ROS accumulation, further exacerbated by Beclin-1-mediated suppression of system X_c_−, reducing GSH and enhancing peroxidation. Signal transducer and activator of transcription 3 (STAT3) further amplifies ferritinophagy via NCOA4 [131]. In cardiomyopathies, such as doxorubicin-induced [132] or diabetic related [133], ferritinophagy-mediated iron release, GPX4 downregulation, and impaired mitophagy collaborate to confer mitochondrial damage and ferroptotic cardiomyocyte death [134].

#### 3.2.5. Ferroptosis and Liver Diseases

Similarly to other major organ-damaging effects, ferroptosis in the liver contributes to various pathologies through a shared axis of labile iron release, antioxidant collapse, and PUFA lipid peroxidation. During hepatic ischemia–reperfusion or toxic injury, NCOA4-mediated ferritinophagy liberates labile iron, amplifying Fenton reactions and membrane damage. Concurrent GSH depletion and GPX4 activity decrease, removing key defenses, while lipoxygenases drive PUFA-phospholipid peroxidation [135]. In metabolic dysfunction-associated steatohepatitis (previously non-alcoholic steatohepatitis), excess lipids and iron synergize to heighten oxidative stress and ferroptotic death [136,137]. Furthermore, lipoxygenase (LOX) isoforms such as ALOX12 catalyze peroxidation of membrane PUFAs, while pharmacological inhibition of ferroptosis, for example, with the plant phytochelator catechin, reduces hepatic inflammation and injury in clinical and preclinical models [15,138,139,140].

In fibrosis and cirrhosis, hepatocyte ferroptosis promotes fibrogenesis, while selectively inducing ferroptosis in hepatic stellate cells can attenuate matrix deposition [141]. Ferritinophagy-driven iron release and altered iron handling (including transferrin-related pathways) further potentiate profibrotic signaling. Increased iron and oxidative environments, such as the ones occurring in hemochromatosis or metabolic dysfunction-associated steatohepatitis, also predispose the liver to carcinogenesis, and modulating ferroptosis, usually with sorafenib, shows therapeutic potential [142], with emerging resistance mechanisms such as upregulated metallothionein-1G (MT1G) [143] and enhanced circTTC13, which suppresses SLC7A11 [144], offering new targets to restore ferroptotic vulnerability in hepatocellular carcinoma [145].

#### 3.2.6. Ferroptosis and Autoimmunity

In addition to its involvement in many diseases, ferroptosis is also increasingly recognized as a driver of autoimmunity by linking iron metabolism, redox imbalance, and immune deregulation. For example, in systemic lupus erythematosus, autoantibodies and interferon-α (IFN-α) suppress GPX4 in neutrophils via cAMP-responsive element modulator α (CREMα), leading to lipid ROS accumulation, neutrophil ferroptosis, and propagation of autoimmunity [146], while iron deposition in kidneys and endothelium exacerbates tissue injury and vascular inflammation [147]. Similarly, in rheumatoid arthritis, synovial cells and chondrocytes exhibit reduced GPX4/SLC7A11 and nuclear factor erythroid 2-related factor 2 (Nrf2) activity [148,149], with ferritinophagy and transferrin receptor protein 1 (TFR1) upregulation elevating labile iron, thereby driving ferroptosis, ROS accumulation, and joint damage [150]. Notably, interleukin-1β (IL-1β) amplifies this axis by repressing GPX4 and promoting NCOA4 [151]. In inflammatory bowel disease (ulcerative colitis and Crohn’s disease), epithelial downregulation of GPX4 and system X_c_− impairs antioxidant defense, rendering intestinal cells susceptible to PUFA-driven lipid peroxidation, barrier breakdown, and chronic inflammation [152]. Importantly, pharmacological inhibition of ferroptosis, for example, with curculigoside [153], reduces inflammation and tissue injury in experimental autoimmune and colitis models [154], highlighting ferroptosis as a therapeutic target in systemic and organ-specific autoimmune disorders.

#### 3.2.7. Ferroptosis and Inflammation

Beyond autoimmunity, ferroptosis also amplifies inflammatory injury in diverse contexts. In chronic obstructive pulmonary disease and cigarette smoke-induced lung injury, iron accumulation and PUFA peroxidation trigger ferroptotic epithelial cell death, releasing damage-associated molecular patterns (DAMPs) and lipid mediators that worsen airway inflammation [155]. In acute respiratory distress syndrome, labile iron increase and LOX-driven PUFA lipid ROS, coupled with GPX4/GSH depletion and impaired Nrf2 signaling, create a vicious cycle where ferroptosis amplifies NLR protein family pyrin domain containing 3 inflammasome (NLRP3 inflammasome) activation and cytokine release (IL-1β, IL-6, tumor necrosis factor-α (TNF-α)), further intensifying oxidative stress and tissue damage [156,157]. Similarly, in stroke, neuronal and glial ferroptosis provoked by iron and ROS not only accelerates cell death but also feeds neuroinflammation through release of damage-associated signals, such as high-mobility group box 1 (HMGB1) and heat shock proteins (HSPs) [158]. Finally, in infection-related inflammation, pathogens exploit iron sequestration dynamics and PUFA oxidation to induce host–cell ferroptosis, aiding immune evasion and persistence [159]. Collectively, these mechanisms position ferroptosis as both an executioner of inflammatory cell death and a trigger of self-perpetuating inflammatory cascades across pulmonary, neurological, and infectious diseases.

In summary, across different organs, systems, and diseases, ferroptosis is unified by iron-driven lipid ROS and reduction in antioxidant defenses, making it both a common driver of pathology and a promising therapeutic target for many diseases (Table 1).

### 3.3. Therapeutic Approaches to Target Ferroptosis

#### 3.3.1. Clinical Agents Under Exploration for Targeting Diseases Associated with Ferroptosis

The targeting of ferroptosis at the clinical level is becoming an important development for the treatment of many associated diseases, and it also increases the prospects for the introduction of a new sector of pharmaceuticals [13,14,15,125]. Promising candidates for the modulation of ferroptosis and the treatment of associated diseases include different drugs, which have been previously shown to have the ability to influence one or more metabolic pathways, factors, or proteins involved in ferroptosis. There are many potential candidate therapeutics with such properties, including drugs with iron chelating, antioxidant, metabolic modulation, or inhibition of pathways or properties associated with ferroptosis, and other drugs or natural compounds targeting inducers of ferroptosis. In each case, the selection of modulating drugs for ferroptosis is subject to many parameters, including risk/benefit assessment, drug posology, and patient characteristics, among others [13,14,15,26,71].

Among the most prominent inhibitors of ferroptosis available for clinical use are the iron chelating drugs DF, L1, DFRA, and the antioxidants vitamin E and N-acetylcysteine [4,13,14]. The efficacy, toxicity, and limitations of use of DF, L1, DFRA, and N-acetylcysteine have been recently reviewed [13,160]. The inhibition of ferroptosis by the iron chelating drugs has been shown in hundreds of experimental non-clinical models of associated diseases, including cancer, neurodegenerative, kidney, cardiac, and other organ damage. These models highlight the importance of the chelating drugs L1 [123,161,162,163,164], DF [165,166,167,168,169], and DFRA [170,171,172,173,174] as potent inhibitors of ferroptosis in associated diseases.

Several other clinically approved drugs have been identified to have secondary effects on ferroptotic pathways, either by promoting or modulating iron-dependent oxidative stress. Although none are yet formally indicated as ferroptosis-targeting therapeutics, their mechanisms link closely with lipid peroxidation, glutathione metabolism, and iron redox cycling. These agents show how repurposing existing drugs can connect ferroptosis research with translational applications, particularly in diseases where oxidative stress and iron deregulation affect pathology. Some of these promising drugs, most of which have been tested for anticancer activity, are discussed below.

Sorafenib, a multikinase inhibitor widely used in hepatocellular and renal cell carcinoma [175], was the first clinically approved compound recognized to induce ferroptosis. Beyond its kinase inhibition, sorafenib blocks system X_c_−, leading to intracellular cysteine depletion, glutathione reduction, and subsequent GPX4 inactivation [176]. This dual activity promotes lethal lipid peroxidation in tumor cells with high metabolic and iron turnover. Preclinical and translational studies have shown that sorafenib sensitivity correlates with the expression of SLC7A11 and iron-handling genes, supporting a link between iron metabolism and treatment response [177]. However, its ferroptotic potency is dependent on the type of cells, and excessive oxidative stress can also promote adaptive resistance through the Nrf2 pathway activation [178,179]. Combination strategies using sorafenib with GPX4 inhibitors, iron chelator complexes, iron-enriched nanoparticles, or immunotherapies are under evaluation to enhance ferroptotic killing of cancer cells while maintaining selectivity.

Artemisinin and its derivatives, including artesunate and dihydroartemisinin, are another class of clinically established agents now recognized to trigger ferroptosis through iron-dependent free radical production. These compounds contain an endoperoxide bridge that reacts with Fe^2+^ to generate ROS, directly linking their cytotoxicity to cellular iron availability [180]. In cancer models, artemisinin derivatives deplete glutathione, increase lipid peroxidation, and downregulate GPX4, leading to ferroptotic death, particularly in cells with elevated labile iron [181]. Early preclinical studies are exploring nanoparticle or transferrin-targeted delivery systems to exploit their anti-ferroptotic cancer potential [182,183,184]. Nonetheless, their systemic use requires careful control of iron levels to avoid oxidative injury in healthy tissues.

Ruxolitinib, a Janus kinase 1/2 (JAK1/2) inhibitor approved for myelofibrosis and graft-versus-host disease [185], is not a direct ferroptosis inducer but can indirectly influence ferroptotic susceptibility by modulating oxidative and inflammatory signaling. Inhibition of JAK/STAT reduces ROS production and Nrf2 activation led by cytokines, which may either protect against ferroptosis [186] or, under certain conditions, such as cancers like osteosarcoma [187], sensitize cells to ferroptosis depending on the redox context [188]. Emerging data suggest that JAK/STAT inhibition can influence iron metabolism genes and glutathione synthesis, indicating potential interactions with ferroptosis pathways in inflammatory and fibrotic diseases [188]. While its main clinical application remains anti-inflammatory, ruxolitinib shows how changes in inflammation and oxidative balance can influence ferroptosis.

Classical chemotherapeutics such as cisplatin and doxorubicin also interact with ferroptotic mechanisms through their effects on iron redox cycling and lipid peroxidation. Cisplatin increases intracellular iron accumulation by impairing ferritin synthesis and iron export, increasing ROS formation, and sensitizing tumor cells to ferroptosis inducers like erastin and RSL3 [187]. Its combination with ferroptosis inducers has shown synergistic cytotoxicity in preclinical cancer models, overcoming resistance regulated by enhanced antioxidant defenses. Similarly, doxorubicin generates ROS via redox cycling of its quinone moiety and forms iron-doxorubicin complexes that catalyze lipid peroxidation, effectively linking oxidative stress and ferroptosis [189]. While these mechanisms contribute to their anticancer activity, major toxicities also arise, most notably cardiotoxicity from doxorubicin, which is increasingly understood to involve ferroptosis of cardiomyocytes [189]. This highlights the need for precise treatments that trigger ferroptosis in tumor cells but protect healthy tissues with inhibitors or iron chelators.

Overall, these clinical examples show that changing iron and redox balance can either trigger or prevent ferroptosis, depending on the tissue and its metabolism. Despite promising early results, using ferroptosis directly as a therapy is still mostly experimental. This has led to new strategies, such as targeted small molecules, genetic tools, and nanomaterials, aimed at controlling ferroptosis more precisely and safely, as discussed below.

#### 3.3.2. Experimental Approaches for Modulating Ferroptosis with Other Potential Therapeutics

A wide range of experimental approaches is under development in addition to modulating ferroptosis selectively through manipulation of iron metabolism and oxidative stress, providing valuable insight into therapeutic design beyond clinically approved agents.

Early small-molecule inducers such as erastin, RSL3, FIN56, and ML210 are classical research tools that directly target core ferroptotic mechanisms. Erastin inhibits the cystine/glutamate antiporter system X_c_−, leading to glutathione depletion and GPX4 inactivation [190], while RSL3 and related compounds irreversibly inhibit GPX4, preventing etoxification of lipid peroxides [191]. Other synthetic agents, including FINO_2_ [192] and iron-salophene complexes [193], promote iron redox cycling and lipid peroxidation by catalyzing Fenton reactions, thereby amplifying intracellular ROS and triggering ferroptosis.

Gene-based strategies have also emerged as powerful experimental tools to regulate ferroptotic pathways. siRNA, shRNA, and CRISPR/Cas9 knockdown of ferroptosis-suppressing genes such as *SLC7A11*, *GPX4*, and *FSP1* markedly sensitize cells to oxidative damage and iron-driven lipid peroxidation [82,194,195]. Researchers are also exploiting metabolic and redox reprogramming to modulate ferroptotic sensitivity. For example, inhibition of glutaminolysis, CoQ_10_ synthesis, or lipid desaturation, such as through SCD1 or ACSL4 modulation, alters membrane composition and enhances susceptibility to iron-catalyzed lipid peroxidation [196,197]. Similarly, suppression of the Nrf2 antioxidant pathway [198] or activation of p53 [199] can shift the intracellular redox balance toward ferroptotic cell death, as also explained above.

Additionally, recent advances in nanomedicine have demonstrated the potential of engineered nanomaterials to induce ferroptosis selectively in cancer cells through modulation of iron metabolism and redox balance. Iron oxide-based nanoparticles (NPs) such as iron-hyaluronic acid [200], polydopamine-polyethylene glycol (PEG) [201], and gallic acid/polyacrylic acid-coated iron oxide [202] have effectively triggered ferroptosis in diverse tumor models by promoting intracellular iron accumulation, GSH depletion, and ROS-driven lipid peroxidation, while sparing normal tissues. Studies have shown that nanoparticle size, surface modification, and encapsulation in liposomes or ferritin shells significantly affect bioavailability, autophagy interactions, and tumor selectivity [203,204,205,206]. Notably, these systems, including liposomal PEG-coated Fe_3_O_4_ NPs [205], ferritin-doxorubicin conjugates [206], and serum-preincubated superparamagnetic iron oxide NPs [207], have demonstrated potent in vivo antitumor efficacy with minimal systemic toxicity, confirming ferroptosis as a viable anticancer therapeutic mechanism distinct from apoptosis and necrosis [208].

Beyond single-function nanomaterials, multifunctional and stimuli-responsive nanoplatforms have been designed to integrate ferroptosis with complementary modalities such as photodynamic therapy, chemodynamic therapy, chemotherapy, and immunotherapy. Systems incorporating pH, GSH, or enzyme-responsive triggers, such as Fe_3_O_4_- poly(lactic-co-glycolic) acid-Ce6 [209], sorafenib@mesoporous polydopamine (PDA)-superparamagnetic iron oxide [210], Fe^2+^/GMP-PDA [211], and MMP-2-cleavable PEG-amorphous calcium carbonate nanocarriers [212], enable controlled release and enhanced ROS generation within the tumor microenvironment. Additional platforms combine ferroptosis with gene regulation or immune modulation, including GAPDH-targeting siRNA co-delivery [211] and miR-21-3p-AuNPs enhancing anti-PD-1 immunotherapy [213], thereby overcoming resistance mechanisms and promoting immune-mediated tumor clearance. Targeted constructs such as endothelin-3-functionalized polymer NPs [214] and salinomycin-gold nanoconjugates [215] further enhance selectivity for tumor or cancer stem cells.

Collectively, these preclinical approaches highlight the versatility of ferroptosis manipulation through, for example, direct GPX4 inhibition, iron redox amplification, and genetic or metabolic interventions. Furthermore, these approaches also serve as essential experimental platforms for defining ferroptosis-specific vulnerabilities and for developing more selective, iron-focused therapeutic strategies in future translational studies.

## 4. Senescence

### 4.1. Oxidative Stress as a Key Player Also in Senescence

Cellular senescence is a state of irreversible cell-cycle arrest triggered by various stressors, including DNA damage, telomere attrition, mitochondrial dysfunction, and oncogene activation. Central to this process are the DNA damage response (DDR) pathways, particularly p53/p21^CIP1^ and p16^INK4a^/Rb, which enforce proliferative arrest [216]. Mitochondrial dysfunction further amplifies senescence by elevating ROS, while senescent cells undergo epigenetic remodeling, formation of senescence-associated heterochromatin foci (SAHF), and loss of lamin B1, stabilizing the senescent phenotype [217].

**Figure 2:**
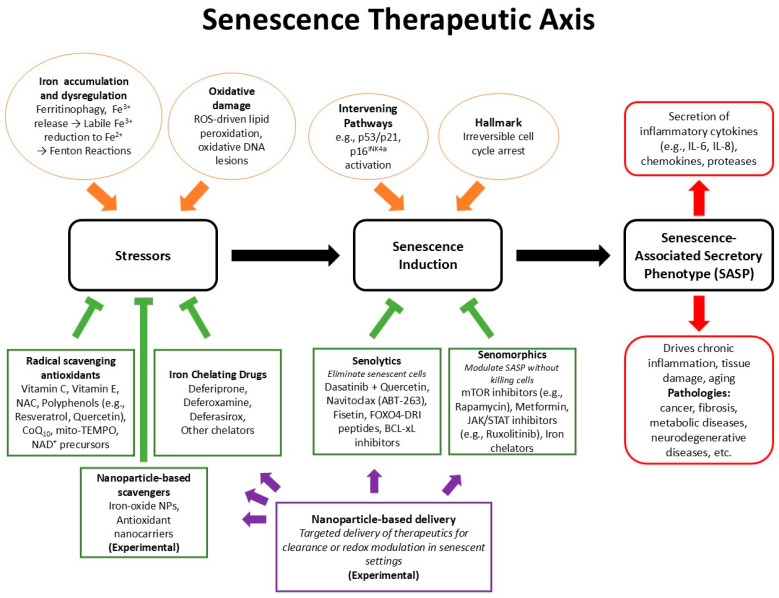
Schematic representation of major pathways driving cellular senescence and their therapeutic targeting. Persistent stressors, including DNA damage, mitochondrial dysfunction, and telomere attrition, trigger p53/p21 and p16/Rb signaling, leading to a type of growth arrest called senescence and its associated phenotype (SASP). Iron accumulation and oxidative stress further reinforce senescence and inflammation. Therapeutic strategies include senolytics (e.g., dasatinib + quercetin, navitoclax, FOXO4-DRI) to eliminate senescent cells, senomorphics (rapalogs, metformin, ruxolitinib) to suppress SASP, and iron chelators or radical scavenging antioxidants to restore redox balance. Nanoparticle-based systems offer enhanced targeting and reduced toxicity for these agents. (Color coding and arrows: Black boxes and arrows depict the core stress–response cascade leading from cellular stressors to senescence induction and the SASP. Orange arrows and outlines indicate upstream stressors and signaling events that promote senescence. Green boxes and arrows represent protective or modulatory interventions targeting oxidative stress and iron dysregulation. Purple boxes and arrows denote experimental nanoparticle-based delivery strategies. Red boxes and arrows highlight SASP-mediated inflammatory outcomes and downstream pathologies. Abbreviations: BCL-xL: B-cell lymphoma-extra large. CoQ_10_: co-enzyme Q_10_. FOXO4: Forkhead box O4. IL-6: interleukin-6. IL-8: interleukin-8. JAK: Janus kinase. mTOR: mammalian target of rapamycin. NAC: N-acetylcysteine. NAD^+^: nicotinamide adenine dinucleotide. NPs: nanoparticles. ROS: reactive oxygen species. SASP: senescence-associated secretory phenotype. STAT: signal transducer and activator of transcription.)

A key feature of cellular senescence is the senescence-associated secretory phenotype (SASP), a complex mixture of pro-inflammatory cytokines (e.g., IL-6, IL-1β), chemokines, proteases, and growth factors, which spreads senescence paracrinally, promotes tissue remodeling, and fuels chronic inflammation. Oxidative stress is both a cause and a consequence of senescence. In particular, ROS induce DNA breaks and protein/lipid oxidation, while ROS-activated NF-κB and mTOR signaling sustain SASP production (Figure 2) [9,218,219].

### 4.2. The Role of Iron in Senescence

Iron metabolism abnormalities profoundly influence senescence by different mechanisms and mainly by exacerbating oxidative stress. In particular, expansion of the intracellular labile iron pool promotes Fenton reactions and hydroxyl radical generation, triggering DNA damage, mitochondrial dysfunction, and lipid peroxidation [12,77]. Senescent cells often accumulate iron due to impaired ferritinophagy and altered iron export, locking them into a redox-imbalanced state that sustains SASP and paracrine senescence [220,221]. Importantly, labile iron redox toxicity also intersects ferroptosis, highlighting mechanistic overlaps between ferroptosis and senescence [222]. Thus, iron metabolism and labile iron redox toxicity emerge as a central link between oxidative stress, senescence, and ferroptosis.

### 4.3. The Interconnection Between Ferroptosis and Senescence

Ferroptosis and cellular senescence are distinct cell stress responses, yet they share common biochemical mechanisms, particularly in relation to iron metabolism and oxidative stress. Both processes are initiated or exacerbated by disruptions in redox homeostasis. In particular, ferroptosis is driven through lethal lipid peroxidation by iron-dependent Fenton chemistry reactions, and senescence through chronic accumulation of ROS that activate DNA damage responses and stress signaling pathways.

Despite this overlap, the outcomes of these processes differ fundamentally. Ferroptosis is an acute, iron-dependent form of regulated cell death, requiring uncontrolled lipid ROS accumulation that overpowers antioxidant defenses such as GPX4. By contrast, senescence is a state of permanent growth arrest in viable cells, typically coupled to adaptations that prevent immediate death, including upregulation of antioxidant systems and iron sequestration mechanisms, such as ferritin induction and altered iron storage/trafficking [223].

Iron handling highlights one of their most critical differences. In ferroptosis, labile iron pools expand, catalyzing lipid peroxidation and membrane destruction. In senescent cells, iron is often retained in excess but compartmentalized and controlled to limit acute toxicity. This iron retention without leading to ferroptosis is thought to contribute to the long-term pro-oxidant environment of senescence, fostering the SASP rather than immediate cell death [220,224].

Thus, while both ferroptosis and senescence are implicated with iron and ROS, their pathways differ, with ferroptosis leading to cell death through uncontrolled oxidative damage, whereas senescence translates oxidative stress into a sustained survival program that reshapes tissue homeostasis. These differences, which are summarized in Table 2, are particularly relevant when considering therapeutic strategies, as promoting ferroptosis may selectively eliminate senescent cells, while controlling senescence-associated iron and oxidative stress may prevent dysregulated tissue remodeling and disease progression.

### 4.4. Diseases Associated with Senescence

#### 4.4.1. Aging

Aging is marked by the progressive accumulation of senescent cells across tissues, where oxidative stress and excess iron deposition accelerate their onset. DNA damage from ROS, telomere attrition, and replication errors engage DDR pathways, while mitochondrial dysfunction amplifies ROS production. Senescent cells contribute to age-related decline by impairing tissue repair, exhausting stem cell pools, and secreting SASP factors that drive inflammaging [225,226,227].

Iron metabolic deregulation and compartmentalized iron overload in specific cells and tissues are crucial mediators of age-related senescence. Increased tissue iron stores with age, observed particularly in the brain, liver, heart, and skeletal muscle [228,229,230,231], heighten oxidative stress and trigger senescence pathways. In the brain, this iron-ROS interplay is linked to cognitive decline [232]; in muscle, to sarcopenia [233]; and in the liver, to the regulation of liver regeneration [234]. SASP-driven chronic inflammation further compromises tissue function and spreads senescence. Animal studies show that clearance of senescent cells restores regenerative capacity and extends health span, underscoring their causal role in organismal aging [235,236,237,238,239]. Further to that, several progeroid syndromes such as Hutchinson–Gilford Progeria Syndrome [240], Werner Syndrome [241], Cockayne Syndrome [242], and Bloom Syndrome [243] share a common hallmark of premature cellular senescence driven by genomic instability and defective DNA repair amplified by oxidative stress.

#### 4.4.2. Diseases Beyond Aging

Senescence contributes to a broad spectrum of chronic diseases, where iron and oxidative stress consistently amplify pathology. Below, there is a short overview of the role of iron, senescence, and oxidative stress in common pathologies beyond aging.

In neurodegenerative diseases there is extensive literature supporting the interplay between oxidative stress, senescence, and neurodegeneration. For example, in Alzheimer’s disease, senescent astrocytes, microglia, neurons, and oligodendrocyte progenitors accumulate, marked by senescence-associated beta-galactosidase (SA-β-gal) activity and increased p16/p21 expression. Their SASP (IL-1β, IL-6, IL-8, and HMGB1) promotes amyloid plaque formation and tau aggregation. Iron deposition in hippocampal and cortical regions exacerbates ROS production, accelerating pathology [244,245]. In Parkinson’s disease, senescent astrocytes and microglia secrete pro-inflammatory SASP, worsening dopaminergic neuron degeneration, while iron overload in the substantia nigra enhances oxidative stress and associated toxicity [246,247]. Similar mechanisms also contribute to amyotrophic lateral sclerosis and Huntington’s disease pathologies [248].

In cardiovascular diseases, endothelial and vascular smooth muscle cell senescence drives atherosclerosis and vascular aging. SASP secretion (IL-1, IL-6, and matrix metalloproteinases (MMPs)) promotes inflammation and plaque instability. Mitochondrial ROS and iron imbalance reinforce senescence, while inflammasome activation (e.g., NLRP3) amplifies vascular inflammation. In myocardial infarction and ischemia–reperfusion injury, senescent cardiomyocytes and fibroblasts promote fibrosis and maladaptive remodeling [249].

In chronic obstructive pulmonary disease, oxidative stress from cigarette smoke induces epithelial senescence via p16^INK4a^/p21 activation. SASP secretion (IL-6, IL-8, and proteases) drives chronic inflammation and paracrine senescence [250]. In idiopathic pulmonary fibrosis, senescent fibroblasts, alveolar epithelial, and endothelial cells accumulate in fibrotic foci, where their SASP (TGF-β and WNT-5a) drives extracellular matrix deposition [251].

In chronic kidney disease, tubular epithelial senescence promotes fibrosis through SASP-driven inflammation. Reactive oxygen species and telomere attrition amplify senescence, while excess iron load exacerbates tubular damage. Persistent senescent cells after acute kidney injury contribute to maladaptive repair and disease progression [252].

In metabolic dysfunction-associated steatotic liver disease/non-alcoholic steatohepatitis, hepatocyte senescence induced by lipid overload, telomere shortening, and oxidative stress promotes SASP secretion (IL-6 and TGF-β), driving inflammation and stellate cell activation. Iron overload in hepatocytes synergizes with ROS to accelerate senescence and fibrosis [253]. In cirrhosis, senescent stellate cells initially restrain fibrosis [254], but their accumulation and SASP release eventually worsen inflammation and scarring.

In osteoporosis, senescent osteocytes and bone marrow mesenchymal stem cells secrete SASP factors (IL-6, IL-8, and TNF-α), promoting osteoclast activity and bone loss. In osteoarthritis, senescent chondrocytes and synovial cells accumulate, releasing SASP (MMP-13, ADAMTS-5, IL-6, and IL-8) that drives cartilage degradation. Iron dysregulation further worsens oxidative damage in joint and bone microenvironments [255,256,257].

Finally, in diabetes, senescent β cells secrete a unique pro-inflammatory SASP (including DUSP3, ING1, KPNB1, IL-1α, IL-1β, and MMPs) that impairs islet function, while senescent adipocyte progenitors promote insulin resistance via SASP-mediated inflammation [258]. Iron accumulation in islets and adipose tissue fuels ROS production, aggravating senescence and metabolic dysfunction [259].

These diverse disease contexts underscore a unifying theme, which broadly suggests that oxidative stress and iron dysregulation initiate and stabilize senescence, making them central therapeutic targets.

### 4.5. Therapeutic Approaches to Target Senescence and Aging

#### 4.5.1. Clinical Agents Under Exploration for Targeting Diseases Associated with Senescence

Therapeutic interventions targeting senescence are rapidly advancing and can broadly be divided into senolytics, which selectively eliminate senescent cells, and senomorphics, which suppress the harmful SASP without killing the cells. These complementary approaches have entered early clinical exploration, with several compounds demonstrating feasibility and biomarker activity in humans. Some of these findings are briefly described in the clinical trials below, where possible interactions with iron metabolism and redox activity are also discussed.

In the senolytics armamentarium, the dasatinib-quercetin (D+Q) combination is the most clinically advanced, with trials, for example, in idiopathic pulmonary fibrosis [260] and Alzheimer’s disease [261] showing feasibility and early efficacy among other encouraging findings [262,263,264]. Similarly, the pilot study in idiopathic pulmonary fibrosis demonstrated feasibility and short-term safety, with participants showing modest improvements in physical function and reduced circulating senescence markers after dosing of D+Q [260]. In Alzheimer’s disease, the SToMP-AD phase 1 trial confirmed central nervous system penetration of dasatinib and observed modulation of senescence-related cerebrospinal fluid biomarkers, again with acceptable short-term tolerability [261]. Additional exploratory work in diabetic kidney disease reported decreases in p16^INK4a^ and p21^CIP1^ expression in peripheral blood cells, suggesting target engagement [262]. Across studies, adverse events were mostly mild and transient (fatigue, gastrointestinal upset, and brief cytopenias), but long-term data remain limited. No consistent alterations in systemic iron indices have been reported in the D+Q trials, although both quercetin and dasatinib possess metal-binding ligands [14], and quercetin itself possesses iron-binding and antioxidant properties that may contribute to its senomorphic actions [14,263,264]. (Examples of clinical trials involving D+Q could be found in NCT05838560, NCT04063124, NCT04685590, NCT05422885, and NCT05653258).

Navitoclax (ABT-263), a BCL-2 family inhibitor, effectively clears senescent cells in preclinical neurodegenerative [265], cardiovascular [266], and metabolic disease [267] models. Clinically, its use has been constrained by predictable, dose-dependent thrombocytopenia, resulting from on-target BCL-xL inhibition in platelets [268]. Early oncology trials established its pharmacokinetics and safety limits, prompting development of intermittent schedules and new BCL-xL-specific inhibitors to mitigate platelet toxicity.

Senomorphic agents target key signaling pathways such as NF-κB, JAK/STAT, and mTOR, which regulate SASP production. Clinically, rapalogs (e.g., everolimus) or other mTOR inhibitors (e.g., RTB101–a TORC1-selective inhibitor) have shown efficacy in improving immune responses in elderly patients, highlighting their potential as senomorphics in aging [269]. More specifically, in randomized trials, low-dose rapalogs enhanced influenza vaccine responses and reduced the frequency of respiratory infections, supporting their potential as anti-aging interventions. Reported adverse events such as mild mucositis, dyslipidemia, and transient hyperglycemia were manageable at geroprotective doses. In general, mTOR inhibitors also appear to influence cellular redox balance but not systemic iron metabolism.

JAK inhibitors, including ruxolitinib, are under evaluation for reducing SASP-driven inflammation in chronic inflammatory and fibrotic diseases and may hold promise for age-related conditions [270]. Clinical trials indicate symptomatic improvement and cytokine suppression in myeloproliferative and dermatological diseases, though infection risk and cytopenias remain dose-limiting toxicities. While their direct impact on iron metabolism appears minimal, JAK inhibition may indirectly alleviate iron-driven oxidative stress by reducing inflammatory cytokines that upregulate hepcidin.

Metformin, widely prescribed for type 2 diabetes, is being repurposed in trials for aging and frailty, for example, the TAME (Targeting Aging with Metformin) trial [271]. Beyond its metabolic actions, metformin attenuates mitochondrial ROS generation and SASP secretion in preclinical models. Observational data link metformin use with reduced incidence of age-related diseases and improved survival in diabetic cohorts. Its safety profile is well established, including gastrointestinal side effects, which are common, and lactic acidosis, which remains a rare event in patients with renal impairment. Although metformin does not directly chelate iron, it reduces redox stress, positioning it as an indirect modulator of iron-driven senescence [272,273,274].

Importantly, several widely used nutraceuticals such as polyphenols and flavonoids, including quercetin alone [275,276,277], curcumin [278,279], and resveratrol [280,281], also act as natural senomorphics by modulating iron homeostasis, scavenging ROS, and dampening SASP signaling. These polyphenols can alter cellular iron handling by increasing ferritin expression or limiting iron uptake in some models [14]. Human trials are generally small and heterogeneous but suggest improvements in inflammatory biomarkers and endothelial function with good tolerability. Examples of clinical trials with quercetin include NCT04907253 and NCT05371340, with curcumin NCT01968564 and NCT03085680, and with resveratrol NCT01126229, NCT01668836, and NCT02123121. Finally, fisetin at lower doses can also act as a senomorphic, suppressing SASP components and attenuating oxidative stress through activation of Nrf2 and inhibition of NF-κB signaling [282], but at higher doses it acts as a senolytic, selectively inducing apoptosis in senescent cells by modulating BCL-2 family proteins and caspase pathways [283]. Beyond its redox activity, fisetin possesses iron-chelating capacity, which may contribute to its antioxidant and anti-senescence effects by limiting iron-catalyzed ROS generation and lipid peroxidation [14]. Examples of clinical trials with fisetin include NCT05025956, NCT04210986, NCT03675724, and NCT03430037. The favorable safety and accessibility of these nutraceuticals make them attractive additions, though pharmacokinetic limitations and lack of standardized formulations remain obstacles to clinical translation [14]. That being said, no senomorphic is yet clinically approved specifically for aging.

Because iron catalyzes the Fenton reaction and amplifies oxidative injury, chelation represents a rational senotherapeutic approach. FDA and EMA-approved iron chelating drugs, including DF, L1, and DFRA, reduce labile iron pools and attenuate ROS-mediated senescence. While primarily used in iron overload syndromes, these drugs are under exploration for senotherapeutic potential in aging and chronic diseases, such as neurodegenerative and metabolic diseases [13,160,284,285].

In more detail, recent studies demonstrate that iron chelation can exert both senomorphic and senolytic effects by restoring iron homeostasis and disrupting the iron-dependent metabolic adaptations of senescent cells. In liver fibrosis, DF and other chelators prevented hepatocyte senescence and fibrotic progression [286], while in osteoarthritis models, DF reduced chondrocyte senescence and oxidative stress [287]. Clinically, DF showed modest neuroprotective effects in Alzheimer’s disease and reduced oxidative markers, though its parenteral route and cumulative toxicity limit its use [288]. Deferiprone crosses the blood–brain barrier and has improved mitochondrial function and reduced brain iron in Parkinson’s disease [289] and Friedreich’s ataxia [290], but outcomes vary by disease context and drug posology [71,291,292,293]. Deferasirox improved hematopoiesis and redox balance in aged animal models [294], though renal and hepatic monitoring remain essential. Overall, iron chelating drugs demonstrate potential to limit iron-driven ROS and senescence, yet their translation to anti-aging therapy requires precise dosing and patient selection to balance efficacy with essential iron-dependent functions and reduce the possibility of toxicity [71].

Finally, there are also other indirect senotherapeutics, including rapamycin [295,296] and the above-mentioned metformin [297,298] which suppress mTOR-driven SASP and restore redox balance, while NAD^+^ precursors [299,300] and caloric restriction mimetics [301] (summarized in [302]) improve mitochondrial function, lower oxidative stress, and reduce senescence burden in preclinical models, although large-scale clinical validation is still pending.

Together, these therapies highlight iron and oxidative stress modulation as central and clinically actionable strategies for managing senescence in aging and different diseases (Table 3).

#### 4.5.2. Experimental Approaches for Targeting Aging and Senescence with Other Potential Therapeutics

There are many experimental approaches for targeting aging and senescence with emerging senotherapeutics, expanding the toolkit beyond approved drugs. For example, the use of FOXO4-DRI peptides has been shown to disrupt the FOXO4-p53 interaction, inducing apoptosis selectively in senescent cells [303,304]. Similarly, the next-generation BCL-xL inhibitors currently under development (including proteolysis-targeting chimera, PROTAC, technology) aim to retain senolytic potency while reducing platelet toxicity, which was observed during a phase-I dose-escalation study of Navitoclax (ABT-263) in lymphoid malignancies [305,306] (Clinical trial number: NCT00406809).

Targeting iron metabolism directly is another frontier for investigations for controlling aging and senescence. In particular, modulating ferroportin, ferritinophagy, or hepcidin pathways may reduce intracellular iron accumulation and oxidative stress in senescent cells [220,228,307,308]. Similarly, targeted mitochondrial antioxidant strategies have been designed, including the mitochondria-specific superoxide scavenger mito-TEMPO [309,310], the mitochondria-targeted antioxidant plastoquinonyl decyltriphenyl phosphonium SkQ1 [311,312], inhibition of minority mitochondrial outer membrane permeabilization (miMOMP) [313], and improved derivatives of known senolytics [314] or novel compounds [315,316,317,318,319]. These and many other approaches are being tested for their ability to restore mitochondrial redox homeostasis and limit SASP, as mitochondrial dysfunction is a key driver of senescence [320]. Nanoparticle-based delivery systems for chelators and senotherapeutics are also under development to increase tissue specificity and minimize off-target effects [321,322,323].

Further investigations are also in progress, including the utilization of immunotherapy strategies against senescence, which is an experimental approach where engineered immune cells or antibodies target senescent cell surface markers for clearance. While at the preclinical stage, such strategies may complement iron and ROS modulation, offering synergistic senotherapeutic potential [324,325].

## 5. Future Prospects in the Treatment of Diseases Associated with Ferroptosis and Senescence

Vast amounts of information are increasingly accumulating in biomedical literature in the last two decades with regard to the causes, development, and occurrence of ferroptosis and senescence, which are implicated in the pathology of many diseases, including cancer, neurodegeneration, infections, organ damage, and aging (Table 1 and Table 3). Such information is critical for understanding both cell processes and for the design of therapeutic strategies, such as those shown in Figure 1 and Figure 2. In the meantime, many clinical trials and experimental approaches with potential therapeutics are being tested for the treatment of diseases associated with ferroptosis and senescence, some with encouraging results but not yet at the stage of full development and regulatory drug approval.

Some of the major and most common features of ferroptosis and senescence include intracellular iron metabolic abnormalities and increased redox toxicity, oxidative stress, and lipid peroxidation. The latter progresses into lipid peroxide aggregates and cell death, which is one of the major hallmarks of ferroptosis but not of senescence, where mainly senescence-associated secretory phenotype (SASP) and DNA damage have been implicated as its cause (Table 2). Further investigations on new targets and targeting methods, kinetic and other differences between ferroptosis and senescence require the design of specific drugs and therapeutic protocols for each disease.

In addition to iron and oxidative stress, all other abnormalities in metabolic, genomic, transcriptional, and other factors during ferroptosis and senescence are also considered as potential targets for therapeutic strategies. In such cases, the aim of any selected therapeutics is expected to cause a clinical improvement or therapy of diseases associated with either ferroptosis or senescence. For example, senomorphic agents are expected to modulate abnormal cellular pathways and could decrease the rate of senescence progression in a disease, whereas senolytic agents could offer complete therapy for such a disease. However, the use of either agents or other potential therapeutics is subject to many investigations and limitations before regulatory drug approval [13,14].

Many toxicity, efficacy, risk/benefit, and other parameters, and targeting issues, apply for the selection of any potential therapeutics for each disease associated with the modulation, inhibition, or induction of ferroptosis and or senescence. For example, access to the affected organs and cells at sufficient concentration levels of a potential therapeutic is one of the critical parameters, which is necessary for targeting. In this context and considering, for example, the use of iron chelating drugs, only L1 can cross the blood–brain barrier and can affect any ferroptosis or senescence targets in the brain [160,326]. Similarly, there is variation in the efficacy of iron removal from different organs in iron-loaded patients, with, for example, L1 being the most effective in cardiac iron removal and DF in liver iron removal [160,327,328]. Furthermore, at the molecular level, all three chelating drugs L1, DF, and DFRA can mobilize intracellular labile iron, but only L1 can remove iron from diferric transferrin, which is partly implicated in the induction of ferroptosis [17,18,329]. Similarly, only L1 and DF have been shown to inhibit lipoxygenase activity and reduce lipid peroxidation [330].

Considering the repurposing of regulatory-approved drugs for the treatment of diseases associated with ferroptosis and senescence, iron chelating drugs and their combinations appear to offer, in general, one of the best therapeutic options for the modulation of iron and the inhibition of Fenton reactions. However, there are toxicity limitations, especially for the use of DFRA and DF in different categories of patients with normal iron stores [13,160]. These limitations do not apply to L1, which has already been tested in many categories of patients with non-iron-loaded conditions such as neurodegenerative diseases, including Parkinson’s, Alzheimer’s, and Friedreich ataxia, kidney damage, cardiomyopathy, infections, cancer, and other diseases [13,331]. In some of these clinical trials, long-term use and high doses have been used. In particular, in one clinical trial, non-iron-loaded Alzheimer’s patients were treated daily with a low dose of L1 (2 × 15 mg/kg/day) for a year and in an HIV short term clinical study much higher doses (150/mg/kg/day), compared to the maximum allowed dose in iron-loaded thalassemia patients (100/mg/kg/day). Most patients did not experience serious toxicity in both trials, and further investigations have been planned [331,332,333].

Drug combination therapeutic strategies involving the targeting of several components are a therapeutic option used in many diseases [159,330]. Considering that both ferroptosis and senescence appear to be multifactorial processes, involving multiple targets, the combination of several drugs may offer more effective treatments. Such combination therapies may include, for example, iron chelating drugs, antioxidants, and lipoxygenase inhibitors for controlling Fenton reactions and lipid peroxidation. In contrast, the therapeutic options may be limited by other treatments or underlying patient conditions. For example, chelating drugs and or antioxidants may cause the opposite effects in anticancer treatment when they are administered simultaneously with iron nanoparticles or iron complexes, which are intended for the induction of ferroptosis in cancer cells [334].

Further information is required for identifying therapeutic targets both in ferroptosis and senescence, and the repurposing of existing drugs or the development of nutraceuticals and new experimental therapeutics that could be used to modulate these cell processes and provide therapeutic solutions in associated diseases [221].

## 6. Conclusions

No therapeutics are yet available for the treatment of many diseases, including, for example, the absence of antioxidant drugs for clinical use in diseases involving oxidative stress and free radical pathologies. However, increasingly new information has been accumulating regarding different aspects of targeting such diseases. In particular, important information has become available in the last two decades regarding the two distinct cell-damaging processes of ferroptosis and senescence, which have been implicated in many diseases affecting millions of people, including cancer, neurodegeneration, organ damage, aging, and other age-related diseases. Such information, and especially the difference in the mechanisms involved and the abnormalities/targets identified between these cell processes, suggests the need for the design of different therapeutic strategies in each case of ferroptosis or senescence involving different drugs and protocols. Accordingly, several repurposed drugs, nutraceuticals, and experimental therapeutics have been investigated in pre-clinical studies and clinical trials for the modulation of ferroptosis and separately for senescence via different targeting methods in each case. Some promising results have been obtained in different disease models of both cell processes, while many clinical trials are still in progress, and new ones have been announced.

A different therapeutic strategy can be adopted regarding the toxicity pathways involving redox iron and oxidative stress damage, which are similar in both ferroptosis and senescence, but with different biomolecular and other toxicity target components in each case and for each disease. Many other limitations apply in the use of potential therapeutics, including combination therapies, all of which are subject to risk/benefit assessment evaluation. It is hoped that many of the investigational drugs could soon become clinically available for the control of both ferroptosis and senescence and for improved therapies in many associated diseases, especially in cancer, neurodegenerative, and other diseases, where no successful treatments are yet available.

## Figures and Tables

**Table 1:** Ferroptosis-related pathways in normal and disease states.

System X_c_− (SLC7A11)	Glutathione Peroxidase 4	Iron Metabolism	Lipid Metabolism	Antioxidants and Redox Regulation	Outcomes
Physiological State					
Regulates cystine import for GSH synthesis and redox homeostasis	Reduces lipid hydroperoxides Prevents membrane damage	Balanced ferritin synthesis and iron storage Regulated ferritinophagy Iron homeostasis maintained by FPN1 export	Normal PUFA turnover Limited lipid peroxidation	Nrf2-related antioxidant enzymes CoQ_10_ regeneration Low ROS	Stable redox balance Controlled iron metabolism Prevention of excess lipid peroxidation
Cancer					
Often ↑ Inhibition enhances ferroptosis	Often ↑ Inhibition enhances ferroptosis	NCOA4-mediated ferritinophagy → ↑ Labile iron pool (LIP) ↑ FTH1/FTL ↓ FPN1	ACSL4 and LOXs promote PUFA-phospholipid oxidation Correlated with ferroptosis sensitivity	↑ Nrf2, FSP1 → antioxidant resistance Inhibition sensitizes tumors to ferroptosis	Baseline ferroptosis resistance Induction of ferroptosis offers a therapeutic strategy
Neurodegenerative Diseases					
Often ↓ in oxidative or inflammatory stress ↓ cystine import → ↓ GSH synthesis	↓ or inactivated → ↑ lipid peroxides in neurons	↑ ferritin ↓ iron export ↑ iron in affected regions → ↑ ROS via Fenton reactions	↑ PUFA oxidation → neuronal death	↓ Nrf2 activity with age ↓ CoQ_10_ ↓ radical scavenging	Ferroptosis contributes to neuronal loss
Ischemia–Reperfusion Injury					
↓ by hypoxia and cytokines → ↓ cystine import and GSH synthesis	Inactivated by oxidative stress GPX4 loss before lipid peroxidation during reperfusion	↑ Fenton reactions due to iron release from ferritin and mitochondria after reperfusion	↑ ACSL4 ↑ lipid ROS in reperfused tissue	Transient ↑ Nrf2 early, but insufficient to prevent ferroptosis When pharmacologically activated → protection	Acute ferroptotic damage → tissue necrosis Ferroptosis inhibition could be protective
Liver Diseases					
↓ System X_c_− → ↓ GSH under stress	↓ or inactivated in hepatocytes	Common iron overload NCOA4-mediated ferritinophagy → ↑ ROS and cell death ↓ FPN1	LOXs and ACSL4 → ↑ PUFA peroxidation	Nrf2 initially enough ↓ Nrf2 under chronic oxidative stress	Ferroptosis contributes to hepatocyte injury and fibrosis Iron chelation might ↓ progression
Acute Kidney Injury					
↓ under oxidative stress	↓ GPX4 → tubular ferroptosis Restoration protects kidneys	Iron accumulation ↑ ferritinophagy → ↑ ROS	↑ ACSL4 expression during injury	When pharmacologically activated → limits damage	Ferroptosis ↑ tubular injury Antioxidants may protect
Cardiotoxicity					
Inhibited by oxidative stress	↓ GSH and ↑ lipid oxidation → GPX4 inactivation	↑ Fenton reactions Impaired ferritin storage capacity	↑ lipid peroxidation → death of cardiomyocytes	Nrf2 pathway protective	Ferroptosis-like death contributes to cardiotoxicity

Abbreviations: ACSL4: Acyl-CoA synthetase long-chain family member 4. CoQ_10_: Coenzyme Q_10_. FPN1: Ferroportin 1. FSP1: Ferroptosis suppressor protein 1. FTH1/FTL: Ferritin heavy and light chain subunits. GPX4: Glutathione peroxidase 4. GSH: Glutathione. LIP: Labile iron pool. LOX: Lipoxygenase. NCOA4: Nuclear receptor coactivator 4. Nrf2: Nuclear factor erythroid 2-related factor 2. PUFA: Polyunsaturated fatty acids. ROS: Reactive oxygen species. SLC7A11: Subunit of the System X_c_− cystine/glutamate antiporter. System X_c_−: Cystine/glutamate antiporter system. ↑: Increase. ↓: Decrease. →: Result.

**Table 2:** Comparative overview of the key biological and therapeutic differences between cellular senescence and ferroptosis.

**Senescence**
(a) Definition: Stable, viable permanent cell-cycle arrest with an active secretome (SASP) that remodels tissue and promotes chronic inflammation.
(b) Kinetics: Chronic, long-lived, develops over days or weeks in response to persistent sublethal stress. Drives long-term tissue remodeling.
(c) Phenotype: Viable but growth-arrested, altered chromatin, enlarged/flattened morphology, SASP secretion (e.g., IL-6, IL-1β, MMPs).
(d) Primary triggers: DNA damage response (p53/p21, p16/Rb), persistent mitochondrial ROS, telomere attrition, oncogene activation.
(e) Iron handling: Senescent cells often accumulate iron through impaired ferritinophagy and altered export, but at the same time compartmentalize iron to avoid immediate ferroptosis, maintaining survival while promoting chronic ROS/SASP.
(f) Lipid metabolism: Altered lipid metabolism contributes to SASP and membrane changes; oxidized lipids can reinforce SASP, but senescence is not primarily executed by lipid peroxidation.
(g) Antioxidant status: Upregulation of some antioxidant responses is common; chronic imbalance sustains SASP.
(h) Pharmacological interventions: Eliminate senescence cells (senolytics) or suppress SASP (senomorphics). Senolytics: D+Q, navitoclax, FOXO4-DRI peptides. Senomorphics: rapalogs, JAK inhibitors, metformin, nutraceutical polyphenols. Iron handling aim: Reduce labile iron to lower ROS and SASP. Agents for iron handling: Iron chelating drugs, such as deferoxamine, deferiprone, and deferasirox, and other chelators, ferroportin/hepcidin modulators, and ferritinophagy inhibitors.
**Ferroptosis**
(a) Definition: Acute, regulated cell death driven by iron-dependent lipid peroxidation and ineffective lipid peroxide detoxification.
(b) Kinetics: Rapid (within hours) once threshold is crossed, relatively fast, lytic death following uncontrollable lipid peroxidation.
(c) Phenotype: Membrane rupture and cell death with vast lipid peroxide accumulation, mitochondrial changes, and release of DAMPs that can be immunogenic.
(d) Primary triggers: Increased labile iron with failure of antioxidant defenses, lipid peroxidation of PUFA-phospholipids, GPX4/GSH axis failure, and FSP1/CoQ_10_ pathway compromised.
(e) Iron handling: Increased labile iron pool catalyzes Fenton reactions, directly causing lipid peroxidation and ferroptotic death. NCOA4-mediated ferritinophagy, the major source of iron from labile iron release and cell sensitization.
(f) Lipid metabolism: PUFA-containing phospholipids are substrates for lethal peroxidation. Lipoxygenases further contribute to enzymatic peroxidation. Lipid composition is central to susceptibility.
(g) Antioxidant status: GPX4/GSH, FSP1/CoQ_10_, or DHODH/BH_4_ pathways are insufficient to detoxify accumulated lipid peroxides.
(h) Pharmacological interventions: Agents for iron handling and modulation: Iron chelating drugs such as deferoxamine, deferiprone, and deferasirox, and other chelators, ferroptosis inhibitors (for protection), iron nanoparticles (NPs), and iron prodrugs (for induction). Iron handling aim: Use iron chelators or ferroptosis inhibitors to prevent death in non-target tissues (for protection). Increase labile iron in targeted cells and/or promote ferritinophagy locally to sensitize cells (for induction). Induce or prevent lethal lipid peroxidation in target cells based on disease settings. Experimental inducers: GPX4 inhibitors (RSL3), System X_c_− inhibitors (erastin analogs), iron-amplifying agents (FINO_2_, iron complexes), and nanomaterials delivering iron.

Abbreviations: SASP: senescence-associated secretory phenotype. IL-6: interleukin-6. IL-1β: interleukin-1β. MMPs: matrix metalloproteinases. Rb: retinoblastoma. ROS: reactive oxygen species. D+Q: dasatinib+quercetin. FOXO4: Forkhead box O4. JAK: Janus kinase. DAMPs: damage-associated molecular patterns. PUFA: polyunsaturated fatty acids. GPX4: glutathione peroxidase 4. GSH: glutathione. FSP1: ferroptosis suppressor protein 1. CoQ_10_: co-enzyme Q_10_. NCOA4: nuclear receptor coactivator 4. DHODH: dihydroorotate dehydrogenase. BH_4_: tetrahydrobiopterin. NPs: nanoparticles. For further information, see references: [11,12,72,75,76,219,220].

**Table 3:** Senescence-related pathways in normal and disease states.

Cell-Cycle Regulators (p53/p21, p16/Rb)	Mitochondrial Function and ROS	Iron Metabolism	Senescence-Associated Secretory Phenotype	mTOR/AMPK Signaling	JAK/STAT Pathway	Antioxidants and Redox Regulation	Autophagy/Mitophagy
Physiological State							
Enable DNA repair Controlled growth arrest during stress	Normal mitochondrial ROS regulate signaling Mitophagy maintains redox balance	Balanced iron storage and export Ferritinophagy recycles iron at a physiological rate	Beneficial signaling in wound healing and regeneration	Maintain energy and growth balance	Regulate immune and cytokine responses	↑ Nrf2 and SIRT1 activity maintain redox homeostasis	Removes damaged mitochondria and other organelles Supports metabolic homeostasis
Cancer							
Chronic activation or mutation p53 loss allows evasion of senescence Therapy-induced senescence contributes to relapse	Oncogenic signaling and hypoxia ↑ ROS Cancer cells often adapt to ↑ antioxidants	Altered iron handling supports proliferation Therapy-induced senescence linked to intracellular iron retention	Tumor-promoting SASP enhances proliferation and immune evasion	↑ mTOR → ↓ autophagy and ↑ SASP AMPK activation restrains tumor growth	IL-6/JAK/STAT3 maintains tumor-promoting SASP Inhibition of inflammation	Often ↓ in tumors or exhausted by ROS	Inhibited by mTOR or oncogenic stress
Neurodegenerative Diseases							
Persistent DDR in neurons and glia → irreversible arrest and neuro-inflammation	Mitochondrial dysfunction → chronic ROS → neuronal senescence-like phenotype	↑ ferritin ↓ iron export ↑ iron → ↑ microglial and neuronal senescence	Chronic SASP → neuro-inflammation and synaptic dysfunction	↑ mTOR ↓ AMPK Metabolic stress in neurons and neuronal degeneration	JAK/STAT activation by cytokines sustains glial senescence and oxidative stress	↓ Nrf2 activity with age → increase in oxidative injury	Impaired mitophagy → accumulation of defective mitochondria
Fibrotic and Metabolic Diseases							
Activated by oxidative and metabolic stress in hepatocytes, adipocytes, and fibroblasts	↑ ROS in liver and fat → insulin resistance and fibrosis	↑ Hepatic iron ↓ ferritinophagy → oxidative stress and fibrogenic senescence	SASP → fibrosis and systemic inflammation	Lipogenesis by mTOR and inflammation worsens disease	Chronic signaling maintains SASP in the liver, kidney, and adipose tissue	↓ Nrf2 and NAD^+^ → hepatic and adipose senescence	↓ autophagy → lipotoxic and fibrotic senescence
Cardiovascular/Other Aging-Related Diseases							
Oxidative stress → endothelial and smooth muscle senescence Telomere shortening	Mitochondrial ROS → endothelial senescence and vascular stiffness	↑ iron in aging tissues → redox imbalance and SASP	Inflammatory SASP contributes to vascular remodeling and atherosclerosis	↑ mTOR → vascular aging	Persistent activation → vascular inflammation	↓ Nrf2/SIRT1 → endothelial dysfunction	Impaired autophagy → vascular stiffness

Abbreviations: AMPK: AMP-activated protein kinase. DDR: DNA damage response. IL-6: Interleukin 6. JAK: Janus kinase. STAT: Signal transducer and activator of transcription. mTOR: Mammalian target of rapamycin. NAD^+^: Nicotinamide adenine dinucleotide. Nrf2: Nuclear factor erythroid 2-related factor 2. Rb: retinoblastoma. ROS: Reactive oxygen species. SASP: Senescence-associated secretory phenotype. SIRT1: Sirtuin 1. ↑: Increase. ↓: Decrease. →: Result.

## Data Availability

No new data were created or analyzed in this study.

## Abbreviations

The following abbreviations were used in the manuscript.

ACSL4	Acyl-CoA synthetase long-chain family member 4
ADAMTS-5	A disintegrin and metalloproteinase with thrombospondin motifs-5
AKI	Acute kidney injury
ALOX12	Arachidonate 12-lipoxygenase
ALOX15	Arachidonate 15-lipoxygenase
BAP1	BRCA1-associated protein 1
BCL-2	B-cell lymphoma-2
BCL-xL	B-cell lymphoma-extra large
BH_4_	Tetrahydrobiopterin
BRCA1	Breast cancer gene 1
CoA	Coenzyme A
CoQ_10_	Coenzyme Q_10_
CREMα	cAMP-responsive element modulator α
CRISPR/Cas9	Clustered regularly interspaced short palindromic repeats/CRISPR-associated protein 9
DAMPs	Damage-associated molecular patterns
DDR	DNA damage response
DF	Deferoxamine
DFRA	Deferasirox
DHODH	Dihydroorotate dehydrogenase
DUSP3	Dual specificity phosphatase 3
Fe	Iron
FOXO4	Forkhead box O4
FPN1	Ferroportin 1
FSP1	Ferroptosis suppressor protein 1
GAPDH	Glyceraldehyde 3-phosphate dehydrogenase
GCH1	GTP cyclohydrolase 1
GMP	Guanosine monophosphate
GPX4	Glutathione peroxidase 4
GSH	Glutathione
GTP	Guanosine triphosphate
HMGB1	High mobility group box 1 protein
4-HNE	4-hydroxynonenal
IFN-α	Interferon-α
IL-1α	Interleukin-1α
IL-1β	Interleukin-1β
IL-6	Interleukin-6
IL-8	Interleukin-8
ING1	Inhibitor of growth family member 1
JAK	Janus kinase
KPNB1	Karyopherin subunit beta 1
L1	Deferiprone
LOX	Lipoxygenase
MDA	Malondialdehyde
miMOMP	Minority mitochondrial outer membrane permeabilization
MiR	MicroRNA
MMP-2	Matrix metalloproteinase 2
MMP-13	Matrix metalloproteinase 13
MT1G	Metallothionein-1G
mTOR	Mammalian target of rapamycin
NADH	Nicotinamide adenine dinucleotide
NADPH	Nicotinamide adenine dinucleotide phosphate
Nrf2	Nuclear factor erythroid 2-related factor 2
NCOA4	Nuclear receptor coactivator 4
NF-κB	Nuclear factor kappa-light-chain-enhancer of activated B cells
NLRP3	NLR family pyrin domain containing 3
NPs	Nanoparticles
·OH	Hydroxyl radical
PD-1	Programmed cell death protein 1
PDA	Polydopamine
PD-L1	Programmed death-ligand 1
PEG	Polyethylene glycol
PROTAC	Proteolysis-targeting chimera
PUFA	Polyunsaturated fatty acid
RNS	Reactive nitrogen species
ROS	Reactive oxygen species
SA-β-gal	Senescence-associated beta-galactosidase
SAHF	Senescence-associated heterochromatin foci
SASP	Senescence-associated secretory phenotype
SCD1	Stearoyl-CoA desaturase
ShRNA	Short hairpin RNA
SiRNA	Small interfering RNA
SIRT1	Sirtuin 1
SLC7A11	Solute carrier family 7 member 11
SOD	Superoxide dismutase
STAT3	Signal transducer and activator of transcription 3
SToMP-AD	Senolytic therapy to modulate the progression of Alzheimer’s disease
TAME	Targeting aging with metformin
TFR1	Transferrin receptor protein 1
TGF-β	Transforming growth factor-beta
TNF-α	Tumor necrosis factor-α
TORC1	Mammalian target of rapamycin complex 1
WNT-5a	Wnt family member 5a
